# The Over-Treatment of Syphilis

**Published:** 1907-04

**Authors:** C. A. Bryce

**Affiliations:** Richmond, Va.


### The Over-Treatment of Syphilis.

BY C. A. BRYCE, M. D., RICHMOND, VA.

Reviewing now not only the literature, but our own long experience in the treatment of syphilis, we are led to ask ourselves a few questions that arise at the present time, and which might have been answered quite differently a dozen or more years ago, in fact, by the majority of teachers even at the present day. One fact must be conceded by all who have kept in touch with current literature relating to this universal trouble, and that is that syphilis is one of the infectious diseases that has certainly not lacked for prolonged, varied and persistent treatment, and that the principal therapeutic agents used have been such as would put a healthy system out of order if subjected to their prolonged influence; and, further, that these agents, pushed to their gradual toxic effects, would induce symptoms and conditions almost identical with many of the prominent symptoms of the disease!

Ignoring in this paper the treatment of the initial stage, when the abortive plan is available and should be practiced, we will consider the management of the disease in its established constitutional form. It does seem a little unscientific and inconsistent in authors and teachers who almost unanimously advise three or four years courses of treatment for all cases of constitutional syphilis, when it is well known to those engaged in daily work in this class of troubles that there are cases that get well without any treatment, some abort, some are well within six months, some in a year, and some vigorously treated for years apparently never get well entirely.

There is something radically wrong in this wholesale injunction to soak and saturate these already blood poisoned patients with mercury, iodine, arsenic, copper, and other drugs, which become irritants and poisons in the system under prolonged use.

If the practitioner would ask himself upon what principle he expected to cure his syphilitic cases, or what he expected to effect with mercury and potass. iodid., and how, whenever he took up a fresh case, he would be getting down to a safe and scientific basis of treatment. Surely, if he administers them with an idea that they are specifics, and to antidote a specific poison, all he has to do is to saturate and antidote and his case is cured—maybe in a month, maybe in five years! If he recognizes, however, that syphilis depends upon cell activity, improved nutrition and elimination aided by *therapeutic doses* of certain recognized agents, he will realize that massive and poisonous doses of so-called specifics tend to lower the patient's powers and aggravate the conditions for which he is being treated.

The great error of the present day in the treatment of syphilis is in overpowering and poisoning the system over long periods with so-called specific remedies. We have seen more direful effects from mercurialization and iodism, pushed, of course, with good intentions, than we have from many cases receiving no treatment at all. Because the system for a time *tolerates* a poisonous dose it must not be inferred that such active poisons are without ultimate deleterious effects that will last possibly for a lifetime! Massive doses of iodide of potash will undoubtedly suppress certain symptoms of constitutional syphilis for a time, but they do more—they do this at the expense of cell integrity, and leave the system a greater sufferer after all. The specialist who studies his individual cases and treats them according to their needs, paying attention to cell building, elimination by skin (especially), kidneys, liver and bowels, general upbuilding of the system and the therapeutic doses of special remedies, will cure his syphilitic patient more speedily, more certainly, and with fewer regrettable accidents than the man who reads his text-books and blindly follows them without observing and thinking that "all coons are not alike" who have syphilis!

We are satisfied that no better introductory to the course of lecture on venereal diseases in our colleges or text-books could be had than full description of the toxic and physiologic effects of mercury and iodine upon the human system. The student and young practitioner should know and become fully impressed with the character of the tools or remedies in his hands. In prescribing them he should do so with a full knowledge of the results and effects they can cause, and he should be able to recognize the manifestation of these agents in certain symptoms produced in his patient instead of being blinded by the supposition that they are the results of a disease he is endeavoring to eradicate with them.

Not a great while ago two of our medical friends referred a prominent gentleman to us for examination and opinion as to the further management of a case of constitutional syphilis. They had treated him faithfully for nine months, and he was getting worse all the time, and had gradually reached a point of rebellion against keeping the same thing up any longer.

He was a man in the prime of life, with apparent good stamina and a willingness of his system to get him well, if allowed a chance. He was suffering with a half-dozen great big copperish-looking carbuncular boils on buttocks, back and leg; he had a foul-smelling breath, soft, bleeding gums and dyspepsia. His attendants had done all that the books suggested, and they had done it faithfully and abundantly. He was taking at the time forty-five grains of iodide of potash daily, and had been doing even better than that for a month or two, with occasional mercurial inunctions to help matters along. Still, he was not getting better, and his physicians had reached a point where they were beginning to do a little thinking in the right direction. They argued that if the man was not improving under all of this treatment, wouldn't it be wise to call a halt and see where they were at?

When we retired for consultation I simply suggested that they should drop the entire idea of syphilis and give the man a chance to get well. Our advice was followed. He was put upon a generous diet of easily digested food, consisting of an abundance of sweet milk, vegetables and cooked fruits. Skin and kidneys were kept active by baths and diuretics, with salines for liver and bowel flushing. A little nux vomica and sarsaparilla to stimulate absorption was the closing treatment and in three months he was a well man—having recovered from drug poisoning—his syphilis ever after remaining an undiscoverable factor, as far as we could see!—*The Southern Clinic.*

